# User Involvement in Developing the MYPLAN Mobile Phone Safety Plan App for People in Suicidal Crisis: Case Study

**DOI:** 10.2196/11965

**Published:** 2019-04-16

**Authors:** Niels Buus, Anette Juel, Hila Haskelberg, Hanne Frandsen, Jette Louise Skovgaard Larsen, Jo River, Kate Andreasson, Merete Nordentoft, Tracey Davenport, Annette Erlangsen

**Affiliations:** 1 Susan Wakil School of Nursing Faculty of Medicine and Health University of Sydney Camperdown Australia; 2 The Centre for Family-Based Mental Health Care St Vincent's Private Hospital Sydney Darlinghurst Australia; 3 St. Vincent's Hospital Sydney Darlinghurst Australia; 4 Department of Regional Health Research University of Southern Denmark Odense Denmark; 5 Psychiatric Research Unit Region Zealand Slagelse Denmark; 6 Clinical Research Unit for Anxiety and Depression St. Vincent's Hospital Sydney Darlinghurst Australia; 7 Mental Health Services. Capital Region of Denmark Copenhagen Denmark; 8 Danish Research Institute for Suicide Prevention Copenhagen Denmark; 9 Psychiatric Research Unit Psychiatric Centre North Zealand Hillerød Denmark; 10 Mental Health Center Copenhagen Copenhagen University Hospital University of Copenhagen Copenhagen Denmark; 11 Brain and Mind Centre University of Sydney Camperdown Australia; 12 Department of Mental Health Johns Hopkins Bloomberg School of Public Health Baltimore, MD United States; 13 Centre for Mental Health Research Australian National University Canberra Australia

**Keywords:** mobile apps, patient participation, primary prevention, self-injurious behavior, suicide, attempted

## Abstract

**Background:**

The effect of safety planning for people in suicidal crisis is not yet determined, but using safety plans to mitigate acute psychological crisis is regarded as *best practice*. Between 2016 and 2017, Australian and Danish stakeholders were involved in revising and updating the Danish MYPLAN mobile phone safety plan and translating the app into a culturally appropriate version for Australia.

**Objective:**

The objective of this study was to examine the negotiation of stakeholders’ suggestions and contributions to the design, function, and content of the MYPLAN app and to characterize significant developments in the emerging user-involving processes.

**Methods:**

We utilized a case study design where 4 focus groups and 5 user-involving workshops in Denmark and Australia were subjected to thematic analysis.

**Results:**

The analyses identified 3 consecutive phases in the extensive development of the app: from phase 1, *Suggesting core functions*, through phase 2, *Refining functions*, to phase 3, *Negotiating the finish*. The user-involving processes continued to prevent closure and challenged researchers and software developers to repeatedly reconsider the app’s basic user interface and functionality. It was a limitation that the analysis did not include potentially determinative *backstage* dimensions of the decision-making process.

**Conclusions:**

The extended user involvement prolonged the development process, but it also allowed for an extensive exploration of different user perspectives and needs.

## Introduction

Mobile apps have the potential to provide evidence-based health care interventions to people who would not otherwise receive services. The major barriers to access include limited resources, geographical location, and poor help-seeking capabilities [1]. Mobile health (mHealth), the use of mobile and wireless technologies to promote health, can assist people in assessing, monitoring, and tracking their mental and physical health; acquiring health information and psychoeducative resources; accessing real-time, recorded, or virtual psychotherapy; and connecting with social networks [2]. However, the potential benefits are counterbalanced by the current limitations to mHealth. Unlike most other health care interventions, the provision of health apps is not highly regulated [3]. There is a plethora of mental health apps, but very little research has explored their efficacy [1,4,5], and there is a growing concern about the effects, usefulness, or potential harmfulness of health apps [6,7].

From the mid-1990s, safety plans have been used in mental health outpatient services as an approach to work with suicidal persons. Without robust evidence, safety planning is considered *best practice* and used as part of different psychological therapies, such as Attempted Suicide Short Intervention Program [8], Cognitive Therapy for Suicide Prevention [9], and Emergency Department Safety Assessment and Follow-Up Evaluation [10]. This has led to some studies regarding the feasibility and acceptability of safety plans [9,11] as well as implementation fidelity and variability [12]. Bryan et al [13] published the first effectiveness study and evaluated the crisis response planning for prevention of suicide attempts. This randomized clinical trial included active duty army soldiers (N=97) presenting an emergency behavioral health appointment. In the trial, the participants received (1) a contract of safety (control group), (2) a standard crisis response plan, or (3) an enhanced crisis response plan. The results from baseline to 6-month follow-up suggest a 76% reduction in suicide attempts and no difference between the enhanced and standard crisis response plan. It was also associated with decline in suicide ideation, fewer inpatient hospitalization days, and larger reduction in negative emotional states [13].

Presently, there is only very limited research on the effectiveness of suicide prevention apps [14,15]. However, 2 published reviews of the design and function of suicide prevention apps provide some insights: Aguirre et al [16] identified 27 apps that could be linked to suicide prevention and assessed them according to (1) research or evaluation of the app, (2) privacy, (3) usability and accessibility, and (4) appropriateness of functionality. In particular, they noted that 12 of the apps did not include a direct link to a crisis hotline. Larsen et al [6] reviewed 49 apps that included at least 1 suicide prevention feature and were available in the Australian Google Play store or the Australian iTunes store. Each app’s features were mapped against 18 suicide prevention strategies and ranked according to these strategies’ level of evidence (from 1 to 4), as identified in the research literature. Only 10 of the 24 suicide-specific apps contained a crisis support or helpline, which was rated as the highest level of evidence, that is, *strong evidence*. Furthermore, the 2 most used app features were peer support (16 apps) and safety planning (13 apps), which were evaluated as having *some evidence* or being *best practice*, respectively. However, it is difficult to make a balanced interpretation of Larsen et al’s findings as they counted the 10 apps available for both Android and iPhone as 20 apps. Both Aguirre et al and Larsen et al concluded that suicide prevention apps need to be supported by stronger research evidence.

In contrast, Nicholas et al [17] made a case for abandoning the traditional evidence base for mobile phone apps, including randomized controlled trials (RCTs). The authors reasoned that the fast-paced mobile market requires alternative and more rapid research evaluation methods, such as iterative participatory research and single case designs. Furthermore, they argued for developing new, alternative ways of accrediting high-quality apps. In accordance with Nicholas et al’s suggestions, we have presented a case study of the iterative user-involving processes that led to the gradual development of the revised version of the mobile safety plan app, MYPLAN [18,19].

MYPLAN was originally a Danish app modeled after Stanley and Brown’s [11] paper-based safety planning tool. The app’s target group includes anyone with a smartphone in, or at risk of, a crisis. This tool combines at least 3 preventive strategies: (1) cognitive, problem-solving, and personalized safety planning (identifying a personal warning sign of an imminent crisis and self-management strategies); (2) encouragement to contact peers and professionals (social support and professional crisis support); and (3) encouragement to limit access to lethal means [11]. The original Danish version of MYPLAN from 2013 also augmented the *encouragement to contact professional support* by including a map with directions to the nearest emergency room. The original version of MYPLAN is currently available in English, Danish, and Norwegian for Android and iPhone [18]. MYPLAN is developed by the Danish MinPlan company.

Democratizing knowledge and ensuring the relevance of research and design to end users are key elements of user involvement [20]. Between 2015 and 2017, the original version of MYPLAN was revised by involving Danish and Australian users with the purpose of developing a cross-cultural adaptation [21] and translation into Australian English. In addition, the revised app’s data storage changed from being app-based to cloud-based, which, for instance, allowed users to share strategies through a Web-based strategy bank (see Table 1 for a brief description of MYPLAN’s key functions in the original and revised app).

**Table 1:** MYPLAN’s key functions in the original versus revised app.

Original MYPLAN	Revised MYPLAN
—^a^	Progressive onboarding: Introductions to the key functions
—	Speed dialing buttons: Customizable speed dialing buttons placed on front page pre-programmed with contact details to emergency services and 24/7 crisis support
—	*Contacts*: Customizable list of personally important contacts
*My symptoms*: Customizable list of personal signs of crisis	*Warning signs*: Customizable list of personal signs of crisis
*Strategies and solutions*: Customizable list of personal strategies for coping with crisis	*Strategies*: Customizable list of personal strategies for coping with crisis
—	*Hope box*: Electronic ‘shoe box’ where personal pictures, videos, and music can be stored and viewed when needed
—	*Mood ratings*: Suicidal Ideation Attributes Scale [22], a psychometric measure of suicidal ideation, and emoji ratings of mood tracked in a calendar
—	*Rant box*: A place where unpleasant thoughts, represented as texts and pictures, can be destroyed by prolonged pressure on the screen
—	*Quick messages*: A space for writing and saving personal text message templates for making quick contact in the future
—	*Share my location*: Sends a text message with the phone’s location using Google Maps
*Nearest emergency*: Directions to the nearest emergency room using Google Maps	*Nearest emergency room*: Directions to nearest emergency room using Google Maps
*Network*: Personally customizable list of important contacts	—
Telephone contact details to 24/7 crisis support	—

^a^Not applicable.

The aim of this study was to examine the negotiation of stakeholders’ suggestions and contributions to the design, function, and content of the MYPLAN app and to characterize significant developments in the emerging user-involving processes.

## Methods

We conducted an instrumental case study [23], which is a useful method for gaining insight into a particular event, such as the user-involving processes that led to the gradual development of the MYPLAN app’s revised design and function.

### Study Context and Participants

Participants were involved in focus groups in Denmark and participatory workshops in Denmark and Australia. The user-involving development process was an opportunistic, emerging design process that began in 4 Danish focus groups. In these focus groups, users suggested that a more interactional setting (workshop discussions between moderators and users) could be beneficial in the ongoing development of the app. Furthermore, the opportunity to develop an Australian English version of the app arose when the first author moved to Australia and 3 workshops were planned across 2 university departments. As outlined below, participants included users, relatives, and clinicians, with the vast majority of participants being users of the app.

**Figure 1:**
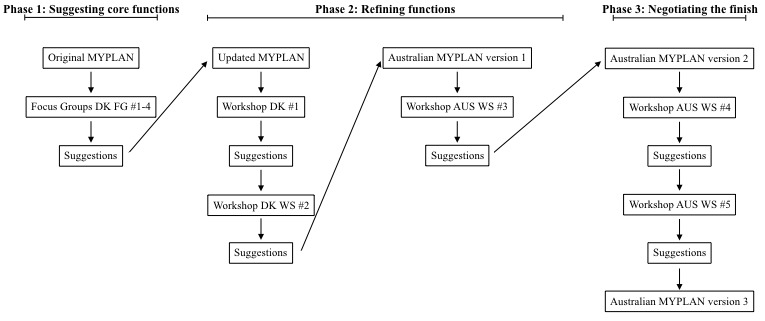
Organization of the app development process.

The focus groups and workshops are detailed in Figure 1 and Table 2.

### Recruitment and Inclusion

In late 2015, 4 Danish (DK) focus groups (FG; DK FG #1-4) were held with key stakeholder participants (adult and young service users, relatives, and clinicians) [19]. All participants were recruited after responding positively to a written invitation distributed by the clinical administrative staff at 2 Danish Suicide Prevention Clinics where the users had received psychosocial treatment for suicidal behavior. The relatives were next of kin of these service users. The focus groups were thematically organized to allow focus on (1) discussing personal experiences of using MYPLAN and (2) participants’ suggestions for improving design and function. In particular, participants were asked to consider whether a safety plan on a smartphone should include auto-generated communication (eg, notifications and prewritten messages), digital memory (eg, a hope box), Global Positioning System (GPS; eg, monitoring), and self-assessment (eg, monitoring and testing). Notable suggestions included incorporating an alarm speed-dialing button, a safety plan for relatives, GPS monitoring, notifications, prewritten messages, a hope box (that could possibly also be shared by a group of users), capacity to share coping strategies, and psychometric tests [19].

**Table 2:** Focus groups and workshops.

Focus groups (FG) and workshops (WS)	Participants	Date	Length (min)	Knowledge of the app
Focus group #1 Denmark: *DK FG #1*	Young users (n=5); mean age: 16.0 years; and moderators (n=2; NB and JLSL)	December 2015	126	Used original version of MYPLAN
Focus group #2 Denmark: *DK FG #2*	Adult users (n=8); mean age: 22.5 years; and moderators (n=2; NB and JLSL)	November 2015	132	Used original version of MYPLAN
Focus group #3 Denmark: *DK FG #3*	Relatives (n=3); mean age: 49.0 years; and moderators (n=2; NB and JLSL)	December 2015	129	Received original version of MYPLAN leading up to the session
Focus group #4 Denmark: *DK FG #4*	Clinicians (n=10); mean age: 46.0 years; and moderators (n=2; NB and JLSL)	November 2015	120	Used original version of MYPLAN
Workshop #1 Denmark: *DK WS #1*	Users (n=3)^a;^ software programmer (n=1); and moderators (n=2; NB and JLSL)	January 2016	155	Used original version of MYPLAN and reviewed wireframes of revised MYPLAN during the session
Workshop #2 Denmark: *DK WS #2*	Users (n=2)^a^ and moderators (n=2; NB and JLSL)	August 2016	156	Used original version of MYPLAN and reviewed wireframes of revised MYPLAN during the session
Workshop #3 Australia: *AUS WS #3*	Users (n=80)^a^ and moderators (n=3; including TD)	August/September 2016	Up to 180	Reviewed wireframes of revised MYPLAN during the session
Workshop #4 Australia: *AUS WS #4*	Users (n=3)^a^ and moderators (n=2; NB and JR)	December 2016	141	Received latest version of revised MYPLAN leading up to the session
Workshop #5 Australia: *AUS WS #5*	Users (n=3)^a^ and moderators (n=3; NB, JR, and HH)	January 2017	188	Received latest version of revised MYPLAN leading up to the session

^a^We did not collect data on these participants’ ages.

In 2016, 2 Danish participatory workshops (WS; DK WS #1-2) were held where end users were invited to evaluate the updated design and function and to suggest further changes to the app. The workshop participants were recruited from participants in the *adult users* focus group, which had been the most active in suggesting changes to the app. The software programmer took part in the first of these workshops.

In 2016, 10 Australian (AUS) participatory workshops (AUS WS #3) were held with 80 participants, including young service users, supportive others, and clinical or service staff from a youth mental health service, headspace. Recruitment for AUS WS #3 was based on inviting potential participants directly through displayed posters and postcards as well as some social media sent out by the participating headspace centers. Over a 3-hour period, the participants examined the wireframes of several apps, including MYPLAN, through an accelerated process of discovery, evaluation, and prototyping (Tracey Davenport, personal communication January 2019).

Finally, in late 2016 and early 2017, there were 2 Australian participatory workshops (AUS WS #4-5) where young service users were invited to evaluate the app for the Australian context. The workshops focused primarily on functions and the culturally appropriate wording of the Australian version, in particular, of the progressive onboarding, which gradually provides users with information when they use the app for the first time. These service users were recruited after responding to a written invitation distributed through Australian mental health services and organizations, such as headspace and ReachOut. All participants were given access to an English prototype of MYPLAN before the workshops.

During the user-involving process, the software developers continually summarized suggestions, developed prototypes, and listed all participant suggestions, which were then evaluated and prioritized with regard to importance and cost.

### Data

As the user-involving processes had an opportunistic emergent design, the available data were heterogeneous. Data from the 4 focus groups (DK FG #1-4) and 4 of the workshops (DK WS #1-2 and AUS WS #4-5) were collected through (1) audio recording of the focus groups and (2) qualitative field notes summarizing suggestions and discussions in workshops. In AUS WS #3, data were collected through (1) written comments made by participants, (2) hand-drawn mock-ups, and (3) qualitative field notes written during the workshops. These latter data were independently knowledge-translated by a representative user team (young people, supportive others, and clinician and service staff) (Tracey Davenport, personal communication January 2019). In addition, written notes produced during the workshops as well as the software developers’ list of suggestions were collected and analyzed.

### Analysis

Data were subjected to thematic analyses [24,25], which included the following: (1) reading or listening to the full dataset to familiarize researchers with content, (2) mapping trajectories of suggestions from inception to rejection or from inception to implementation, (3) parallel coding of full dataset and written summaries by 2 researchers, (4) gradual development of descriptions of thematic content and discrete phases, and (5) corroboration of the description of phases by re-examining data.

### Ethics

We notified the relevant Danish regional research ethics committee and the Danish Data Protection Agency about the Danish focus groups and workshops (DK FG #1-4 and DK WS #1-2); neither institution reported any reservations toward the study. The University of Sydney Human Research Ethics Committee approved the research of Australian participatory workshops (AUS WS #3: reference #2016/529 and AUS WS #4-5: reference #2016/749). All participants gave their informed consent to participate based on written and oral information about the study. Interview responses were handled in full confidentiality, and all details that could potentially be used to identify individual participants have been altered in the data extracts presented in the Results section below.

## Results

### Overview

The analysis identified 3 temporal phases during the user-involving processes, which were characterized by distinct types of negotiations. The first phase, *Suggesting core functions*, was characterized by a focus on discussing the potential inclusion of basic app features. The second phase, *Refining functions*, was characterized by testing and negotiating the design of newly implemented app features. The third phase, *Negotiating the finish*, was characterized by tests and discussions about the final layout and wording. The 3 data extracts presented in the sections below were selected because they were characteristic of the different phases.

Discussions in all 3 phases were characterized by very low levels of displayed disagreement. For instance, when different opinions were voiced, participants would most often resolve disagreement by suggesting that a given app feature should ultimately be optional and adaptable by individual users. Tables 3 and 4 illustrate the different phases with 2 examples, speed dialing buttons and mood rating.

The development and implementation of the speed dial buttons was gradual and characterized by minor edits of the software developer’s responses that were only slightly different from what users had originally suggested.

The development and implementation of the mood rating was gradual but characterized by reluctance from the users regarding its usefulness, in particular, the Suicidal Ideation Attributes Scale (SIDAS) questionnaire. The texts introducing and describing SIDAS scores were completely rewritten by users before final implementation.

### Phase 1: Suggesting Core Functions

The first phase primarily took place during the Danish focus groups, where the functions of the original MYPLAN app were discussed along with ideas for new functions, which were introduced by the researchers. Participants raised some principal issues about the app’s design and functions:

First, some participants voiced different understandings of what users might enter into the app’s core problem-solving function: *Warning signs*. They noted that the concept was unclear and could be interpreted as either *signs of a potential crisis* that could be used in an early intervention to avoid a crisis or as *signs of a current acute crisis* that could be used to mitigate an ongoing crisis. While in phase 1, these discussions did not lead to explicit suggestions for changes to the app; the discussions reappeared in phase 3 with regard to whether the app should be used as a safety plan or suicide prevention tool (see below).

**Table 3:** Examples of the phases in the user-involvement processes: speed dial buttons.

Phase 1: Suggesting core functions, DK focus groups #1-4	Between phase 1 and phase 2	Phase 2: Refining functions, DK workshops #1 and #2 and AUS workshop #3	Between phase 2 and phase 3	Phase 3: Negotiating the finish, AUS workshops #4 and #5	After phase 3
Users suggested speed-dialing buttons. The colors of the buttons should reflect *the degree of emergency* (yellow or red). They should be simple to use: an emergency call should be made with no more than 2 clicks	Development of wireframes with different types of menus and different opportunities for placing an alarm button	The location of the 2 buttons was discussed and they were placed at the bottom of the front page. The yellow button should be named *Help* and was assigned a telephone icon. The red button should be named *Alarm* and was assigned an exclamation mark icon. The way to assign contacts to the buttons was discussed and the number of possible contacts to assign to the yellow button should be 10	Implementation of 2 buttons on the front page. A yellow *Help* button with a telephone icon and a red *Emergency* button with a white exclamation mark. Alarm call in 2 clicks from front page (but a 4-digit access code was also added). A maximum of 10 *yellow* contacts was implemented	The need for better onboarding information, explaining the way to add contacts to buttons, was noted. The icon on the yellow button should be a telephone and a head in a circle. The icon on the red button should be a white cross in a red circle	Onboarding information about how to assign contacts to the 2 buttons was discussed. Implementation of 2 buttons on the front page. A yellow *Help* button with a telephone icon and a red *Emergency* button with a telephone icon

**Table 4:** Examples of the phases in the user-involvement processes: mood ratings.

Phase 1: Suggesting core functions, DK focus groups #1-4	Between phase 1 and phase 2	Phase 2: Refining functions, DK workshops #1 and #2 and AUS workshop #3	Between phase 2 and phase 3	Phase 3: Negotiating the finish, AUS workshops #4 and #5	After phase 3
Users discussed the usefulness of tests and here the idea to use emojis for the mood rating arose. In general, users argued against using tests in the app. Suggestion of making mood rating customizable	Introduction to different types of emoji-based mood trackers	The selected emojis were reviewed and edited (they looked angry rather than sad). The wording of the mood tracking function was discussed and changed. Suggestion of clear introductory text. The function of mood rating, including its reminders, should be customizable	Implementation of emoji-based mood tracker and SIDAS^a^, suicidal ideation measure. The presence of mood ratings in the menu became customizable by individual users	Objection to the wording of SIDAS’s response categories. Introductory text to SIDAS is reformulated. The feedback from SIDAS should be gentler and less demoralizing	Implementation of revised onboarding information about SIDAS and its response categories

^a^SIDAS: Suicidal Ideation Attributes Scale [22].

Second, users were aware not to overcomplicate the app by suggesting too many functions, which occasionally happened when participants started outbidding each other with numerous interactive Facebook-inspired features. For participants, the focus on *need to have* functions rather than *nice to have* functions was related to concerns about developing an app that could be used in an emergency where simplicity would be paramount.

In the following data extract from focus group DK FG #4 (see Textbox 1), which included relatives of MYPLAN users, the participants discussed the potential use of GPS. The prompt for the discussion was a vignette about a young girl who switched on her GPS when she felt suicidal, which would automatically alert parents and clinicians, who would then be able to follow her phone’s location. After expressing concerns that MYPLAN users might be bluffing and that there would be a need for sincerity from the users, the relatives started reflecting on the personal costs such alerts would have on themselves as well as issues of intrusions that GPS surveillance might inflict on their sons or daughters.

**Data extract 1.**

Moderator: *I hear you describing something like the existing ‘Find My iPhone’ feature that parents use to check if the kids are at school or other places. What are your thoughts?*

Emma: *No. That would not be OK for me. Ian [her son] would definitely be too old for that, so I would find that it was intruding on his private space.*

Jenny: *That would send Rachel [her daughter] round the bends; you would be stalking her, right? I think it is good, you could use it in crisis situations or that they can use it in crisis situations.*

Emma: *It must not turn into surveillance, because they will just switch it off.*

Rachel: *It has to be optional.*

Moderator: *So it would be ok if you had an agreement with your daughter for a while?*

Rachel: *Yes, if I could cope with it, my daughter would have to control it herself.*

Emma responds to the moderator’s question by refusing the idea of monitoring her adult son, as it would be too intrusive. Jenny follows up by agreeing that it would not be good for her daughter, but she adds that it might be good in a crisis. She revises her own initial statement when she describes GPS monitoring as something “you” could use in a crisis situation, to something “they” (their sons and daughters) could use, which could indicate that she preferred to describe it as the offspring’s tool and not the parents’. Emma continues by voicing a similar concern and emphasizes that it must not have a character of surveillance as that would be unacceptable and the children would simply not use it. Rachel emphasizes that it has to be optional for the children to be followed by GPS and concludes by stressing that it would require an agreement and that her daughter would have to control it. However, she also states that she, herself, would need to be able to cope with being able to monitor her daughter, which could indicate that it would be emotionally taxing to have access. The option of GPS seems to trigger these participants’ ambivalence about, on one hand, having control and certainty and, on the other hand, the anxiety of a hands-off approach to their vulnerable children.

In phase 1, the researchers ultimately controlled the discussions as they introduced the focus group agenda highlighting the core app features. However, participants were invited and most often able to voice concerns that had a direct impact on the development of the first draft designs of the core functions.

### Phase 2: Refining Functions

The second phase primarily took place during workshops DK WS #1-2 and AUS WS #3 after the software developers and programmers had created a first revision of the app. The majority of app changes were only available as printed wireframes, and discussions were focused on developing additional ideas.

As in phase 1, there was a continual flow of suggestions that were extensively negotiated in the groups as well as suggestions that were never discussed in any significant depth because of their apparent complexity (eg, when you speed dial the emergency services, the phone should automatically send your GPS coordinates or the phone should be able to prompt you or your network if you come near certain risky locations according to data you have entered yourself, for instance a bar or a tall building). Only 1 new core function was developed for the app in this phase. This was the Rant Box, which was introduced following the AUS WS #3. The Rant Box was a place where self-selected text and images could be destroyed by applying prolonged pressure to the touch screen.

In workshops DK WS #1 and 2, relatively structured discussions organized by the moderators took place. The moderators closed the workshop by restating the key points raised and decisions made. In DK WS #1 and 2, moderators both implicitly and explicitly drew on insights from the focus groups. Points were mostly discussed by stating ideas one after the other without much explicit disagreement. There were several instances in DK WS #1 where the participating software programmer funneled the discussions in a particular direction based on arguments linked to the concrete programming of features and economy. Finally, there was an incident where the software designers chose to keep a feature, the structured (SIDAS [22]) suicidality rating scale, despite repeated pushback from user participants who perceived it as redundant and unnecessary (see Table 4). The software developers’ motives for keeping SIDAS despite the users’ pushback were not clear in the available data as the decision, or the lack of a decision, was made in *backstage* negotiations away from focus groups and workshops.

The following data extract 2 (see Textbox 2) illustrates the typical collaborative nature of negotiating and refining a function during a workshop. The participants were discussing different ways of designing a speed-dialing function and had previously settled on a solution with 2 buttons at the bottom of the home screen, a yellow for subacute situations and a red for acute crisis. Now, the key issue was to discuss how much flexibility a user had in terms of assigning particular functions to each of the buttons. Interactions were fast, and in the data extract, “…” indicates that the next speaker started talking by interrupting the previous speaker slightly.

**Data extract 2.**

James: *My idea would be that it [the red button] would do only one thing. When you press the red one then you call a particular person or 112 [emergency call].*

Linda: *I think that too. Alternatively, you could, if you wanted both 112 and Lifeline, then you could add two…*

Moderator 1: *Or you made 112 the red [button] and Lifeline the yellow [button].*

Linda: *Yes, yes, then you’d…*

James: *Yes, of course…*

Moderator 1: *I believe that the red one should be—only need one click. You shouldn’t have to make any decisions…*

James: *That’s also what I think. The yellow is meant for when you feel terrible and you need to talk to someone. When I have been so far out that I simply needed to talk to someone. That would be how I saw the yellow phone [button] whereas the red phone is when I’m almost out of reach. I need to get hold of someone before things go really wrong. Therefore, as you say, the red one is for one person whereas with the yellow you might choose between several. There needn’t be a maximum, but between five and six persons. Having 20 persons to choose from should not confuse you.*

James starts out by explaining his ideas about the differences between the buttons’ functions, and Linda follows up by explicitly agreeing but, at the same time, adding that the red button could be linked to several numbers. The moderator disagrees by emphasizing that he believes that the red button should be simple to use. James expresses his agreement with that and expands on his idea for the 2 buttons by explicitly drawing on his personal experiences of being in a crisis. He concludes by rewording the issue regarding simplicity that had previously been forwarded by moderator 1.

The workshops were highly interactive, with moderators being actively engaged. The researchers had a high degree of control through their ongoing engagement and a systematic and structured summarizing, which ultimately funneled a consensus about each discussion point. However, the very explicit and consensus-seeking approach gave participants a direct influence on decisions. The individuals who had participated in both a focus group and the workshops seemed to display more ownership of the process and had a better understanding of what was being developed, which strengthened and qualified their contributions in discussions.

### Phase 3: Negotiating the Finish

The third phase primarily took place during workshops AUS WS #4 and 5 when MYPLAN’s core functions had been designed, tested, and refined. Fixing glitches continued to take place as it had been done in phase 2, with all issues listed and fed back to the software developers to prioritize and resolve.

Unlike the Danish participants, Australian user participants were not introduced to the app by health care professionals who they had met in the clinic. Therefore, the Australian workshop participants relied heavily on the built-in electronic introductions (e-introductions), which they did not find intuitive or helpful. The Australian participants identified a need for better e-introductions and suggested progressive onboarding to MYPLAN’s functions. The participants from workshops AUS WS #4 and 5 were engaged in writing and editing the introductory texts for the app, both during the workshops and via email after the workshops. These texts were sent to the software developers, who implemented them in both the Australian and Danish versions. Later, the text introductions were supplemented by video clips.

The Australian participants felt that the text introductions, which were relatively noninteractive, made the app’s particular clinical language use unacceptable. The following data extract 3 is taken from AUS WS #4 where a participant, Sarah, highlighted her discomfort concerning the image and text she read after responding to the 5 items of the SIDAS:

It says the word suicide a lot and it says a lot of words that probably when you are in distress you don’t need to be confronted with. And more importantly, it is talking about and not to you again, which is really kind of demoralizing. I feel like it could be a lot shorter and more pleasant very easily.

Sarah

Sarah continued to elaborate on her position that the language use came from a medical and paternalistic position, which, to her, objectified the user and was not helpful for a person in a crisis. This led to a series of discussions about the general coherency: was it a problem that MYPLAN was, on one hand, a personalized self-help tool and, on the other hand, employed medical surveillance and medical psychoeducation? The Australian workshop participants strongly supported the development of a completely nonmedicalized and nonpathologized self-help safety plan, asserting that this would be acceptable for people who felt distressed but who would not identify as being in a crisis (a crisis management plan), let alone being suicidal (a suicide prevention plan).

The pushback from the Australian workshops was a genuine surprise for the researchers who were forced to reconsider their own core assumptions about the app’s purpose and its users. Nevertheless, they appreciated the feedback and welcomed the user participants as authors of the app’s onboarding texts and features.

## Discussion

### Principal Findings

The case study identified 3 temporal phases during the user-involving processes, which were characterized by distinct types of negotiations: (1) *suggesting core functions*, (2) *refining functions*, and (3) *negotiating the finish*. The phases most probably reflected that the MYPLAN app was developed gradually over an extended period of time. Researchers controlled most of the concrete user-involving processes, but as stakeholders were presented with increasingly finalized revisions of the app, they were able to grasp new designs and engage more collaboratively with researchers and software developers.

Models of user involvement often classify levels of involvement according to the distribution of power and control between user participants and researchers [26]. For example, Hanley et al [27] differentiate among the following: (1) *Consultation*, where service users’ perspectives are explored by researchers and potentially brought in to decision-making processes; (2) *Collaboration*, where researchers are actively committed to engaging in ongoing partnerships with service users; and (3) *User control*, where “the locus of power, initiative, and subsequent decision making is with service users rather than with the professional researchers” [27]. Although the user-involving processes analyzed in this study clearly included elements of *consultation*, it remained debatable to what extent the software developers and researchers were committed to *collaborate* and genuinely share control. For example, the software developers were adamant in maintaining the SIDAS despite user dissent. In addition, although study participants were consulted over extended periods of time and occasionally designed direct suggestions to the app, which resembled elements of *user-controlled* involvement, the researchers ultimately controlled the data-collection sessions and the information that was recorded, prioritized, and fed back to the software developers and programmers.

Although users are regularly involved in the evaluation, design, and development of mental health apps (see for instance [28,29]), the actual levels of involvement remain challenging to ascertain. We were, at times, surprised by the users’ nonconsensus-seeking language and their multifaceted, nuanced, and layered suggestions that were not as distinct as we had anticipated, and it was hard to pinpoint when *a suggestion* had been made. This, in effect, made it impossible to track the historical trajectories of distinct suggestions, and it was not possible to identify a specific level of user involvement. The identification of distinct levels of user involvement would most probably rely on preplanned and highly structured negotiation processes that could inadvertently silence the users’ voice. Finally, difficulties in ascertaining user involvement are complicated by the fact that there is no agreed terminology for describing *user involvement* practices. Terms such as co-design, coproduction, and co-development often imply that users are only being *consulted*, that is, not involved as a resource in their own right, and continue to have very little actual power and control in health research and development. We were not able to identify any study on actual user involvement in the research and development of mental health apps. Hence, more research in this area is needed to determine the actual levels of involvement and the benefits of involvement.

It was also difficult to gauge if and how the user-involving processes enhanced the MYPLAN app. Hawton et al’s [30] Cochrane review suggested possible mechanisms as to how the preventive strategies implemented in the app might mitigate suicidal crisis. These include enhancing problem-solving and coping skills (achieved by linking warning signs and strategies) and an increased sense of social connectedness (achieved by listing social contacts and quick messages). Although Stanley and Brown’s [11] paper version of the safety-planning tool drew on at least 3 different preventive strategies, the original as well as the revised MYPLAN apps added further features, in particular, by making use of smartphone technology (telecommunication, digital memory—for instance, the personal hope box or the shared inspiration bank—automatic communication, psychometric testing, and GPS). Moreover, control was handed over to users in the workshops’ discussions, which led to surprising (for researchers) suggestions, for example, the use of nonpathologizing and clinical crisis language. In line with the general aims of user-involving strategies, these processes were perceived to be relevant by users but added complexity to the designing process, and it remains unclear whether the actual and proposed changes could have unintended negative consequences on the design of the app.

In addition, when compared with a paper version of a safety plan, the numerous preventive strategies added to the app could hypothetically decrease transparency and user-friendliness and obstruct *simple* safety planning. O’Toole et al [14] identified the potential negative effects of the use of a multifunction suicide prevention strategy and suggested that this could be related to users having to learn to use new technology (LifeApp’tite) and being prompted to self-rate on a daily basis. Interestingly, many of the functions that O’Toole et al listed as potentially having an adverse effect were functions that users in this study objected to and which led to the design of MYPLAN as a demedicalized crisis management app with comprehensive onboarding recourses. It is possible that the involvement of end users can assist app developers, clinicians, and researchers in developing mHealth technology that remains simple and relevant to users.

As noted by Grundy et al [3], adverse events and possible harm are rarely mentioned in disclaimers of mental health care apps. However, no reports of harm were voiced during any of the sessions and participants’ reports of potential ambivalence or adversity were managed in situ and in the ongoing design of the app. Grundy et al [3] also noted that mental health care apps have a tendency to claim easy and rapid improvement of mental health in their presentations in the app store. However, contrary to these visible and positive claims, the formal app disclaimers tended to distance themselves from presenting the app as a medical service [3]. Arguably, this happens to clearly and conveniently differentiate a given app from a *medical device*, which would be subjected to extensive—and expensive—medicolegal regulation. Regulation would, of course, be necessary if an app intends to be used for diagnosis, prevention, monitoring, treatment, or alleviation of medical diseases. However, phase 3 of this study’s user-involving process included a strong push toward demedicalizing the management of everyday feelings of distress, rather than preventing suicide, which could make the app relevant to a much wider audience. This, *in effect*, moved the app away from potentially being classified as a medical device. To some extent, the revised design of the app begs the question of whether there might be a need for a parallel version of the app that was a medical device and had a strong explicit focus on suicide prevention.

### Strengths and Limitations

We collected observational data from a range of meetings over a long period of time, which allowed a basic mapping of the introduction and negotiation of ideas over time. However, we did not have observational data of the separate negotiations with the app designers. Such negotiations included discussions among software developers about which of the listed suggestions should be prioritized in light of their complexity and costs and decisions taken by the app programmers. From a methodological perspective, we believe that users’ involvement might be perceived as less impactful if these *backstage* negotiations were part of the analysis. Arguably, users should be formally involved in as many of these crucial design processes as possible to achieve more genuine collaboration.

Most participants were offered to join several sessions, which allowed them to voice their opinion as the app was developed gradually over time and to strengthen their sense of personal ownership. However, despite elaborate recruitment strategies, most workshops included only a very limited number of participants. Although we held several workshops and most participants had strong voices and opinions, it would have been advantageous to recruit larger and more diverse groups of participants.

### Conclusions

The analyses identified 3 consecutive phases in the extensive development process of a safety plan provided as an app. Although the phases reflected a gradual implementation process, the user-involving processes continued to prevent closure and challenged researchers and app developers to continually rethink basic app design and functions. The implementation process of the MYPLAN app will aim to continually implement further items from the list of suggestions, subject to available resources. This includes monitoring the use of the specific functions and omitting the ones that are not being used.

Reconsidering Nicholas et al’s [17] introductory argument for abandoning RCTs in evaluations of health apps, it seems that iterative participatory research and single case design (similar to the user-involving processes analyzed in this study) allow for intuitive new innovations. However, such processes cannot evaluate the long-term impacts of apps; evaluating these effects would require different and more extensive methods of testing, such as RCTs [7]. The Danish revised version of MYPLAN is currently being tested against a *nonsmart* safety plan written on paper in a randomized trial [31].

The variety of mHealth tools are likely to increase globally, which highlights a need for procedures for safe adapting, translation, and tailoring of apps across countries and cultures. In line with Harper Shehadeh et al [21], we believe that detailed reporting of adaption methods is crucial, and the systematic involvement of service users could be an important way to increase the trustworthiness of such adaptions.

## Abbreviations

e-introduction: electronic introduction
GPS: Global Positioning System
mHealth: mobile health
RCT: randomized controlled trial
SIDAS: Suicidal Ideation Attributes Scale
